# Ceylon's Hospitals

**Published:** 1906-12-22

**Authors:** 


### CEYLON'S HOSPITALS.

According to the annual report on Ceylon just published, there were at work in the island during 1905 sixty-five hospitals and asylums, 424 Government dispensaries, and 142 estate dispensaries. The statement of expenditure shows that the cost last year of hospitals and dispensaries controlled by the State was 1,323,673 rupees (or, at the rate of legal tender in Ceylon, £88,244), against 1,274,473 rupees (£84,964) in 1904. But the total expenditure on medical institutions amounted to 1,809,585 rupees (£120,639), while the revenue collected from medical sources was 119,563 rupees. The cost of the medical department rose from 402,633 rupees in 1904 to 443,998 rupees in 1905. With regard to the progress of the year the report states:—

"A new hospital built of stone and with the latest improvements was opened at Dikoya, with accommodation for eighty-five patients, and the building of a new hospital at Dolosbage was commenced. The Victoria Memorial Eye Hospital, with accommodation for forty-two patients, was opened in August, and is supplied with the latest equipment. Modern aseptic furniture and instruments have also been supplied to some of the older hospitals."

In-patients in 1905 numbered 68,321 in all hospitals and asylums, of whom 6,697 died, or 9.8 per cent. At Government dispensaries 1,222,790 new cases were treated during the year, which is considerably over a quarter of the whole population. It is interesting to note that ninety-nine senior students and twenty-four apothecary students were on the books of the Ceylon Medical College.

